# Simultaneous Accelerated Corneal Crosslinking and Laser *In situ* Keratomileusis for the Treatment of High Myopia in Asian Eyes

**DOI:** 10.2174/1874364101812010241

**Published:** 2018-08-27

**Authors:** Jin Rong Low, Li Lim, Jane Chwee Wah Koh, Daniel Kai Peng Chua, Mohamad Rosman

**Affiliations:** Singapore National Eye Centre, 11 Third Hospital Avenue, Singapore 168751, Singapore

## Simultaneous Accelerated Corneal Crosslinking and Laser *In situ* Keratomileusis for the Treatment of High Myopia in Asian Eyes

The Open Ophthalmology Journal, **2018**, 12: 143-153

The below mentioned values in Figure 2 have been removed.

**Post-operative 6-12 months= 0.33,0.79 and 0.13**

The below mentioned text has been added in Figure 5.

**% of eyes within + 0.5 D of attempted correction**

